# The importance of being heterozygote: effects of *RHD*-genotype-sex interaction on the physical and mental health of a non-clinical population

**DOI:** 10.1038/s41598-021-00977-1

**Published:** 2021-11-09

**Authors:** Jaroslav Flegr, Lenka Příplatová, Jana Hlaváčová, Blanka Šebánková, Emanuel Žďárský, Šárka Kaňková

**Affiliations:** 1 Department of Philosophy and History of Science, Faculty of Science, Charles University in Prague, Viničná 7, 128 44 Prague, Czech Republic; 2 +DPrevence, Department of DNA Prevention, Prague, Czech Republic

**Keywords:** Evolution, Genetics, Physiology, Diseases, Risk factors

## Abstract

Human populations, especially European, are polymorphic in the *RHD* gene. A significant fraction of their members carry no copy of the coding section of *RHD* gene, which results in their Rh-negative blood type. Theoretically, this polymorphism should be unstable. Carriers of the less frequent allele are penalized by reduced fertility because of the immunization of RhD-negative mothers by their RhD-positive babies, which results in hemolytic disease of the fetus and newborn in their subsequent progeny. For about 90 years, some form of balancing selection has been suspected to sustain this polymorphism. Several recent studies showed that the RhD-positive heterozygotes express higher viability than both types of homozygotes. However, the genotype of subjects in these studies was estimated only by indirect methods. Here we compared the physical and mental health of 178 women and 86 men who were directly tested for their *RHD* genotype. The results showed that RhD-positive homozygotic women had worse and RhD-positive homozygotic men better physical health than RhD-negative homozygotes; the difference between RhD-negative homozygotes and heterozygotes was not significant. Our results confirmed that health of RhD-positive heterozygotes and homozygotes differ. Therefore, any result of the comparison of subjects with RhD-positive and RhD-negative phenotype depends on the heterozygote-to-homozygote ratio in the RhD-positive sample. It is, therefore, crucial to analyze the effects of *RHD*-genotypes, not phenotypes in future studies.

## Introduction

About 1% of native people in East Asia, 5% of those in Africa, and 16% of those in Europe have an RhD-negative blood type^1^. “While RHDΨ is the most frequent allele in RhD-negative Africans^2^, in Europe, 95% of RhD-negative individuals carry two copies of the mutated allele of the *RHD* gene with all translated and most transcribed parts deleted. Such individuals, as well as individuals with other deletions in the *RHD* gene, therefore lack the most dominant D epitope on the surface of their erythrocytes^3,4^. RhD-positive subjects carry either one or two unmutated alleles of the gene.

Mainly for historical reasons, the individuals with one wild allele and one allele with the large deletion are usually called heterozygotes. They could be also called hemizygotes, however, as all coding parts of *RHD* gene are deleted in most of RhD-negative Europeans. We will use the term heterozygote throughout the text as the superiority of the individuals with only one copy of the wild gene^5^, as well as different responses of these individuals to infection with *Toxoplasma*^6^, that the alleles with all DNA between two Rhesus boxes deleted have still some specific effects on the phenotype of their careers. It is possible that the variant or the zygosity of some regulatory element in the 5′ flanking part of the gene is responsible for the observed phenotypical effect of the “deleted gene”. Another possibility, the effects of adjacent genes, is discussed below.

The main role of Rh complex is to transport NH_3_ or CO_2_ gases and their ions^7,8^ but the physiological role of this transport remains unknown. For a long time, the stable coexistence of two alleles of the gene has been an evolutionary riddle. Before the discovery of modern prophylaxis, carriers of the rarer phenotype, either RhD-negative women in a predominantly RhD-positive population or RhD-positive men in a predominantly RhD-negative population, expressed lower fecundity because part of their latter-born babies (RhD-positive babies born to RhD-negative mothers) died of hemolytic disease of the fetus and newborn^9,10^.

During the past 15 years, several studies demonstrated that *RHD* polymorphism is sustained in the population by balancing selection, namely by the selection in favor of heterozygotes. One group of studies showed that RhD-positive heterozygotes have better reaction times than RhD-positive and negative homozygotes when latently infected by the protozoan parasite *Toxoplasma gondii*^6^. Currently, only about one-third of the population is infected with this parasite and the prevalence is decreasing at the rate of about 1% per year in most developed countries^11,12^. However, it is highly probable that the majority of people were infected in our recent evolutionary past implying that we are optimally adapted to being *Toxoplasma *infected. Later, an ecological study showed that frequency of heterozygotes negatively correlates with the incidence of many diseases and with morbidity of other diseases assessed based on DALY (Disability Adjusted Life Years). The study controlled for five potential confounding variables: GDP, latitude (distance from the equator), humidity, medical care expenditure per capita, and frequency of smokers^13^. Other evidence of the effects of RhD phenotype on human health were obtained in several cross-sectional studies. The most detailed study performed on 3130 subjects showed that RhD-negative subjects scored significantly worse in 6 of 22 ordinal health-related variables than RhD-positive subjects^14^. The results also showed that RhD-negativity was positively associated with the incidence of 21, and negatively with the incidence of 10 of 154 diseases under study. However, most cross-sectional studies compared the health and performance of subjects with RhD-positive and RhD-negative phenotypes and all relied on the information provided by participants of anonymous internet studies. The only exception is a recent study performed on 2539 subjects^5^. In this study, respondents were asked not only about their RhD phenotype but also RhD phenotype of their biological parents. This enabled the identification of a substantial part of heterozygotes—RhD-positive subjects with an RhD-negative mother or father. The study showed that RhD-positive heterozygotes have better health than RhD-negative homozygotes and also brought very strong evidence that they have better health even in comparison with RhD-positive homozygotes.

The main purpose of the present study is to confirm the effect of *RHD*-genotype on physical and mental health using a non-anonymous study with subjects whose *RHD*-zygosity was determined by the direct molecular method in a laboratory. For this purpose, we analyzed the physical and mental health-related data of 178 women and 86 men, the representatives of the nonclinical Prague population, who consented to participate in the study performed at the Faculty of Science, Charles University.

## Results

The population under study consisted of 178 women and 86 men. Men (mean age: 32.3, SD = 10.3) were about 3 years older than women (mean age: 29.1, SD = 10.3), t = − 2.45, p = 0.015, Cohen d = 0.32. Among women, we detected 43 (24.2%) RhD-negative homozygotes, 52 (29.2%) RhD-positive homozygotes, and 83 (46.6%) RhD-positive heterozygotes. Among men, we detected 24 (27.9%) RhD-negative homozygotes, 23 (26.7%) RhD-positive homozygotes, and 39 (45.3%) RhD-positive heterozygotes (*RHD* genotype-sex: χ^2^ = 0.466, df = 2, p = 0.792).

**Figure 1:**
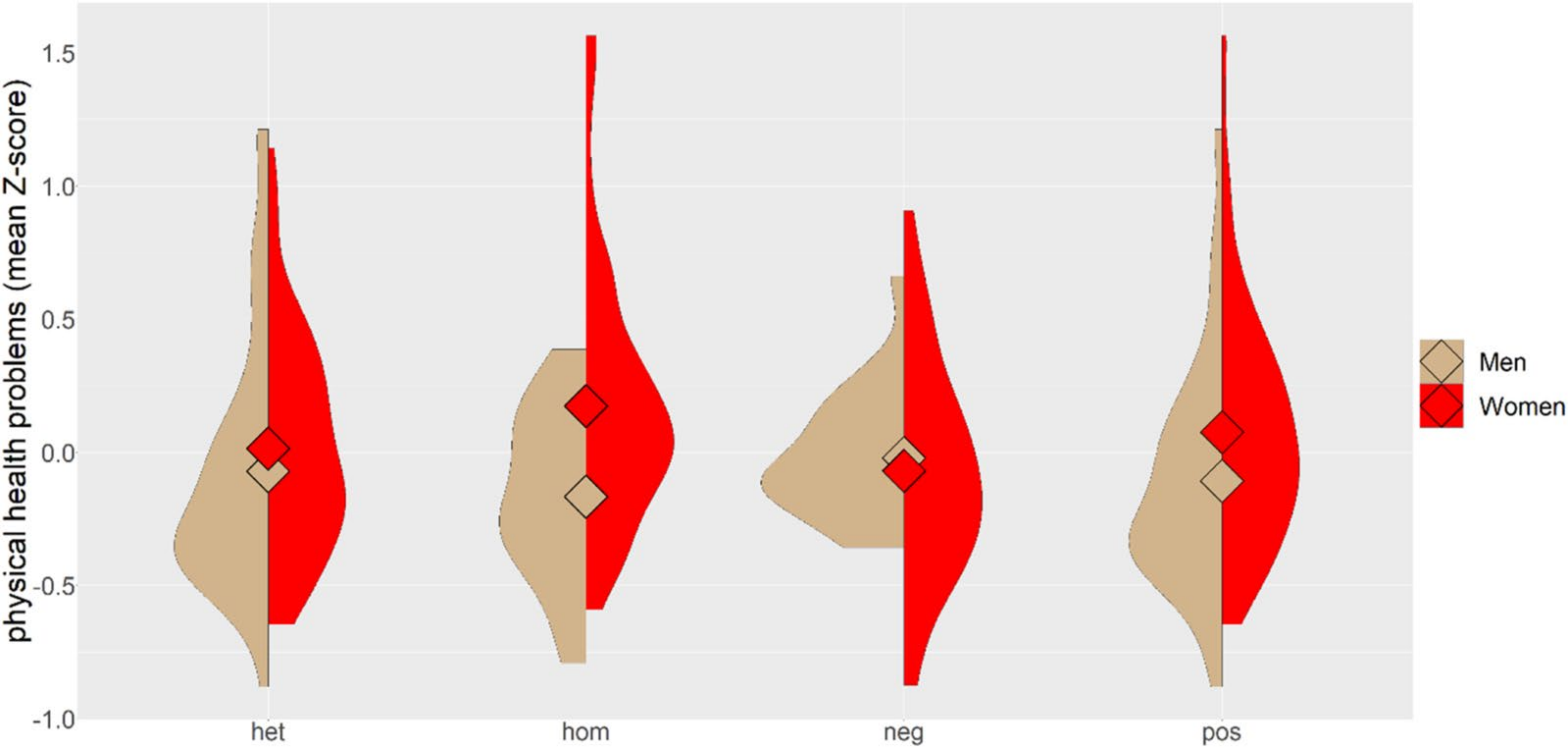
Physical health problems score of men and women with different *RHD* genotype. The figure shows the means and the distribution computed as the kernel probability density for male and female heterozygotes (het), RhD-positive homozygotes (hom), RhD-negative homozygotes (neg), and RhD positive participants (pos).

The ANCOVA test with *physical health problems score* or *mental health problems score* as the dependent variable, sex and *RHD* genotype as factors, and age as a covariate found a significant negative association of age with *physical health problems score* (β = − 0.172) and *mental health problems score* (β = − 0.235), as well as a significant association of RhD genotype-sex interaction with the physical health problems score, see the Table 1. Visual inspection of Fig. 1 showed that RhD-negative homozygotes had similar physical health as RhD-positive heterozygotes, however, male RhD-positive homozygotes have the best, and female RhD-positive homozygotes the worst physical health of all six groups. The shape of the distributions also suggested that subpopulations of heterozygotic women and RhD-positive homozygotic men might be heterogenic—they could contain two distinct populations. Also, the physical health problems score of heterozygotic men might be negatively affected by a small number of outliers with very bad health. Separate ANCOVA tests showed that the association between *RHD* genotype and *physical health problems score* was significant for 178 women (p = 0.039, η^2^ = 0.035) but not for less numerous (86) men (p = 0.429, η^2^ = 0.020). The association of *RHD* genotype with the *mental health problems score* was neither significant for women (p = 0.225, η^2^ = 0.017), nor for men (p = 0.795, η^2^ = 0.006).

**Table 1:** Effects of *RHD* genotype on physical and mental health measured with ANCOVA.

	Physical health problems score			Mental health problems score		
	F	p	η^2^	F	p	η^2^
Age	7.980	0.005	0.030	14.695	0.000	0.054
Sex	3.565	0.060	0.014	0.071	0.789	0.000
*RHD* genotype	0.133	0.876	0.001	1.189	0.306	0.009
Sex-*RHD* genotype	3.299	0.038	0.025	0.078	0.925	0.001

Post hoc ANCOVA tests with *physical health problems score* as the dependent variable performed separately for women and men showed significantly worse health in RhD-positive female homozygotes than RhD-negative female homozygotes (p = 0.026, η^2^ = 0.053, β = − 0.225) and RhD-positive female heterozygotes (p = 0.047, η^2^ = 0.030, β = 0.170). No other difference between *RHD* genotypes was significant either for physical or mental health problems score.

To see which specific health problems were responsible for the observed effect of the *RHD* genotype, we performed a partial Kendall correlation test controlled for age for all source variables originally used for computing physical and mental health problems scores. Table 2 shows that, generally, heterozygotes have better health than RhD-positive homozygotes. In women, RhD-negative homozygotes had better health than RhD-positive homozygotes and often also than heterozygotes while the opposite is true for men. However, the general pattern also had some exceptions. For example, heterozygotic men scored better in most parameters of physical and mental health than RhD-positive homozygotes but they reported suffering from more chronic problems and consuming more medical drugs currently.

**Table 2:** Effects of *RHD* genotype and phenotype on physical and mental health problems scores and their source variables.

	All				Women				Men			
	** + −/++**	** + −/− -**	**++/− −**	p + /p-	** + −/++**	** + −/− −**	**++/− −**	p+/p**−**	** + −/++**	** + −/− −**	**++/− −**	p+/p**−**
Physical health problems score	− 0.080	0.003	0.086	0.031	**− 0.129**	0.059	**0.194**	0.098	0.004	− 0.158	**− 0.209**	**− 0.156**
Mental health problems score	**− 0.123**	− 0.027	0.095	0.017	**− 0.122**	− 0.008	0.117	0.035	− 0.120	− 0.079	0.045	− 0.031
Acute illness in the last month	0.001	0.073	0.076	0.064	0.025	0.078	0.058	0.062	− 0.052	0.049	0.113	0.067
Acute illness in the past 6 months	− 0.070	− 0.023	0.045	0.003	0.019	0.055	0.047	0.046	**− 0.295**	**− 0.218**	0.072	− 0.097
Chronic problems treated now	0.011	**0.115**	0.104	**0.097**	− 0.058	0.108	**0.161**	**0.110**	**0.175**	0.138	− 0.046	0.070
Chronic problems all	0.001	0.056	0.061	0.052	− 0.067	0.031	0.094	0.048	0.160	0.102	− 0.030	0.052
Drugs prescribed	0.055	0.080	0.043	0.057	− 0.017	0.107	0.132	0.097	**0.217**	0.041	− 0.145	− 0.020
Bad physical health today	− 0.043	− 0.081	− 0.058	− 0.063	− 0.111	− 0.086	0.021	− 0.040	0.103	− 0.061	**− 0.214**	− 0.102
Headaches	− 0.059	0.052	0.111	0.066	**− 0.127**	0.068	**0.190**	**0.102**	0.074	− 0.032	− 0.106	− 0.054
Migraines	− 0.033	0.078	**0.115**	0.078	− 0.100	0.084	**0.186**	**0.104**	0.161	0.068	− 0.147	0.014
Life expectancy	0.062	− 0.028	− 0.115	− 0.052	0.060	0.059	− 0.013	0.031	0.083	− 0.173	**− 0.344**	**− 0.217**
Allergies	0.038	0.003	− 0.022	− 0.007	0.002	0.018	0.035	0.022	0.129	− 0.012	− 0.136	− 0.056
Skin disorders	**− 0.131**	− 0.072	0.053	− 0.022	**− 0.167**	− 0.119	0.054	− 0.048	− 0.036	0.039	0.049	0.038
Circulatory system disorders	− 0.018	0.013	0.027	0.017	− 0.064	− 0.034	0.040	− 0.002	0.071	0.074	0.000	0.050
Blood pressure	0.001	− 0.082	− 0.075	− 0.070	− 0.048	− 0.049	− 0.009	− 0.028	0.131	− 0.146	− 0.191	− 0.150
Digestive tract disorders	0.051	0.062	− 0.003	0.037	0.027	0.091	0.051	0.070	0.106	− 0.045	− 0.164	− 0.080
Metabolic disorders	0.060	0.089	0.026	0.060	0.064	0.108	0.048	0.076	0.024	0.011	− 0.036	− 0.003
Orthopedic disorders	− 0.075	0.060	**0.142**	0.081	**− 0.131**	0.085	**0.222**	**0.119**	0.024	− 0.068	− 0.062	− 0.056
Neurological disorders	**− 0.112**	− 0.006	0.097	0.031	**− 0.155**	0.020	**0.177**	0.070	− 0.019	− 0.050	− 0.064	− 0.051
Headaches 2	**− 0.099**	0.066	**0.186**	**0.097**	− 0.098	**0.140**	**0.249**	**0.159**	− 0.121	− 0.128	0.041	− 0.059
Physical pains	**− 0.132**	− 0.005	**0.126**	0.038	**− 0.177**	− 0.006	**0.171**	0.050	− 0.080	− 0.034	0.072	0.007
Chronic physical problems	**− 0.205**	**− 0.102**	0.113	− 0.020	**− 0.306**	− 0.077	**0.249**	0.038	0.008	− 0.160	− 0.193	**− 0.161**
Tired	− 0.073	− 0.048	0.020	− 0.020	− 0.073	− 0.010	0.060	0.015	− 0.044	− 0.159	− 0.083	− 0.118
Tired after work	**− 0.134**	− 0.094	0.040	− 0.041	**− 0.170**	− 0.112	0.063	− 0.043	− 0.054	− 0.074	− 0.014	− 0.047
Tired after train	− 0.052	0.045	0.080	0.052	− 0.051	0.072	0.111	0.079	− 0.040	− 0.027	0.017	− 0.003
Tired after bus	**− 0.171**	0.001	**0.155**	0.051	**− 0.189**	− 0.039	**0.146**	0.027	− 0.106	0.053	0.122	0.076
Common infectious diseases	**0.098**	− 0.049	**− 0.141**	− 0.076	0.091	0.098	0.013	0.059	0.106	**− 0.357**	**− 0.429**	**− 0.348**
Medical doctors	− 0.074	0.026	0.099	0.048	− 0.106	0.062	**0.171**	0.091	0.013	− 0.056	− 0.062	− 0.047
Antibiotics in the last year	0.053	0.074	0.052	0.058	0.045	**0.146**	**0.143**	**0.127**	0.068	− 0.090	− 0.140	− 0.102
Antibiotics in the last 3 years	− 0.014	0.012	0.034	0.017	− 0.039	0.061	0.129	0.076	0.060	− 0.093	− 0.158	− 0.106
Hospital in the last year	**0.119**	**0.116**	− 0.017	0.073	0.112	0.095	− 0.025	0.055	0.121	0.142	NA	0.100
Hospital in the past 5 years	− 0.003	**0.140**	**0.152**	**0.125**	− 0.020	**0.127**	**0.162**	**0.119**	0.031	0.161	0.136	0.138
Bad mental health today	**− 0.119**	− 0.028	0.080	0.011	**− 0.163**	− 0.007	**0.161**	0.050	− 0.017	− 0.084	− 0.102	− 0.079
Learning disabilities	− 0.005	0.050	0.052	0.045	0.001	0.085	0.083	0.073	− 0.020	0.016	0.037	0.021
Mentally bad today	− 0.041	− 0.026	0.017	− 0.010	− 0.079	− 0.045	0.044	− 0.012	0.032	0.016	− 0.038	− 0.002
Mentally bad usually	**− 0.178**	− 0.093	0.085	− 0.025	**− 0.167**	− 0.074	0.104	− 0.007	**− 0.198**	− 0.125	0.063	− 0.053
Anxiousness	− 0.016	− 0.020	0.002	− 0.010	− 0.024	− 0.026	0.006	− 0.011	− 0.010	− 0.031	− 0.072	− 0.045
Depressiveness	**− 0.097**	− 0.045	0.048	− 0.010	− 0.077	− 0.066	0.006	− 0.034	− 0.133	− 0.012	0.114	0.032
Phobiaes	0.039	− 0.009	− 0.057	− 0.023	0.037	0.001	− 0.041	− 0.011	0.011	− 0.070	− 0.107	− 0.074
Depressions	**− 0.132**	− 0.057	0.064	− 0.010	− 0.099	0.016	0.106	0.045	**− 0.202**	**− 0.211**	− 0.002	− 0.122
Other mental health problems	**− 0.106**	0.074	**0.183**	**0.101**	− 0.080	0.101	**0.182**	**0.114**	− 0.168	0.018	0.196	0.077

This table shows the direction and strength of particular effects (Taus) measured with a nonparametric partial Kendall correlation test controlled for age. The++, + **−**, **− −**, p**−**, and p+ at the column headings denote RhD-positive homozygotes, RhD-positive heterozygotes, RhD-negative homozygotes, RhD-negative and RhD-positive subjects, respectively. Negative Tau means that the genotype or phenotype that is on the left from the slash has a better health parameter than the genotype that is on the right from the slash. Significant Taus (p < 0.05) are printed in bold. The results that remained significant even after the correction for multiple tests with the Benjamini–Hochberg procedure are underlined.

To be able to compare our results with those already published, we pooled RhD-positive homozygotes and heterozygotes and repeated the analyses with the binary variable Rh-positivity instead of nominal variable *RHD* genotype (Fig. 1). ANCOVA tests showed no significant effect of RhD phenotype or RhD phenotype-sex interaction on the scores of mental or physical health problems (physical health-sex-Rh: p = 0.103, η^2^ = 0.010; mental health-sex-Rh phenotype: p = 0.912, η^2^ < 0.0001). However, partial Kendall analyses showed several significant effects of RhD phenotype on the physical and mental health-related variables. Generally, RhD-positive women reported worse and RhD-positive men better health than their RhD-negative peers (Table 2 (Taus) and Supplementary Table 1 (p-values)).

## Discussion

Our results suggest the effect of *RHD* genotype-sex interaction on physical health. Generally, RhD-positive homozygotic women reported worse physical health than both RhD-negative and heterozygotic women, while RhD-negative men reported worse physical health than RhD-positive homozygotic and heterozygotic men. Similar yet nonsignificant trends in the same direction are present when the mental health of subjects with RhD-negative and RhD-positive phenotype is compared. Better health of heterozygotes than homozygotes was expected a priori based on the theory and already published data^5^. Still, we used more conservative two-sided tests in all parts of the study. Result of this test, the effect of RhD-sex interaction on physical health, remained significant (p = 0.019) after the correction for multiple (here two) tests.

Detailed analyses showed that RhD-positive homozygote women reported being more often tired, more often spending more than 7 days in a hospital in the past 5 years, having more frequent chronic health problems, physical pain, headaches, migraines, orthopedic problems, neurological problems, attending medical doctors more often, taking more antibiotics in the last year, and having “other mental health problems” more often than RhD-negative homozygote women. In contrast, RhD-positive homozygote men reported better physical condition at the time of blood sample-taking, a higher life expectancy, and less frequent common infectious diseases than RhD-negative homozygote men. The number of significant effects was lower in men than in women, probably because of the much lower number of men than women in our sample (86 vs 178), however, the effects were usually stronger than in women. For example, a partial Kendall’s Tau -0.429 for common infectious diseases was equivalent to R^2^ 0.49, which means that *RHD* genotype was responsible for nearly 50% of the variability in the frequencies of common infectious diseases in the sample of male RhD-positive and negative homozygotes.

A comparison of female RhD-positive homozygotes and heterozygotes showed that heterozygotes had fewer headaches, skin disorders, orthopedic disorders, neurological disorders, suffered less physical pains, less chronic physical problems, are less frequently tired, and feel in better mental health conditions usually, as well as at the time of blood sample-taking. The same comparison for male homozygotes and heterozygotes showed that heterozygotes had had fewer acute illnesses in the past 6 months, feel usually in better mental conditions, and especially feel less depressed. At the same time, heterozygotes reported to be treated for chronic problems more often—which corresponded with taking more prescribed drugs at the time of blood sample-taking.

Heterozygotic men reported less frequent acute disorders, especially common infectious diseases, and less frequent or less serious depression than RhD-negative men. Paradoxically, heterozygotic women reported more frequent headaches, consuming more antibiotics, and staying for more than 1 week in the hospital more often than RhD-negative women. A higher frequency of headaches in (healthier) heterozygotes was already described in the previous study^5^.

A comparison of women with RhD-positive and RhD-negative phenotype showed worse health of the former. RhD-positive women reported more chronic problems, more headaches, migraines, orthopedic disorders, more frequent use of antibiotics in the past year, more frequently spending at least 7 days in a hospital in the past 5 years, and more intensive “other” mental health problems (other than depression, anxiousness, phobias). RhD-positive men reported longer life expectancy, less frequent chronic physical health problems, and less frequent common infectious diseases. In fact, RhD-positive men scored non-significantly better in nearly all other health-related variables (except frequency of hospitalization) but most of these (sometimes relatively strong) associations were non-significant because of the low number of male participants in the study.

Present results can be compared with those of the recent study based on data from 2539 respondents of an electronic questionnaire (23% Rh negative)^5^. In this study, a subpopulation of RhD-positive heterozygotes was identified based on their Rh-phenotype (positive) and Rh-phenotype of their biological parents (either father or mother RhD-negative). This design did not allow comparing RhD-positive homozygotes with the other two groups because part of heterozygotes did not report parents’ RhD phenotype and these subjects finished in the same group as homozygotes. Moreover, the spectrum of health-related variables under the above-mentioned study was different and far narrower in comparison with the present study.

Results of the internet study^5^ showed that heterozygotes have better health than both types of homozygotes. In contrast to the present study, subjects with RhD-positive phenotype (especially men) expressed better health than those with RhD-negative phenotype, which is in agreement with the results of other studies. The internet study found a stronger effect of Rh phenotype on mental health than on physical health; in the current study, the effect of genotype on mental health was not significant. Besides, the previous study observed worse physical health in RhD-positive than in RhD-negative homozygotes both in men and women (not only in women as it was in the current study). Nevertheless, it must be remembered that the current study was performed on just 86 men (23 RhD-positive and 23 negative homozygotes) compared to 502 men in the previous study. Therefore, the absence of certain significant effects could be the result of the smaller population sample analyzed in the current study.

Another recent internet study performed on a sample of 5527 participants (24% RhD negative) compared only the physical and mental health of subjects with RhD-positive and RhD-negative phenotype^15^. This internet study found worse health in RhD-positive women and better health in RhD-positive men in comparison to corresponding RhD-negative controls.

All available data about the performance of RhD-positive homozygotes and heterozygotes^5,6^ and the present study therefore suggest that RhD-positive heterozygotes have better and RhD-positive homozygotes have worse heath than corresponding RhD-negative subjects. Consequently, it could be argued that the results of a study depend on the heterozygote-to-homozygote ratio among RhD-positive subjects. This ratio increases with the increasing prevalence of RhD-minus allele in the population under study, which depends not only on its prevalence in a general population but probably also on the auto-selection of the participants of the study—see the over-representation of RhD-negative subjects in the studies discussed below.

Another biological variable that should be taken into consideration is the prevalence of latent toxoplasmosis in studied population sample. This prevalence varies approximately from 10 to 70% among various countries, depending on environmental conditions (especially moisture), eating and other cultural habits, and hygienic standards^11,12^. It also strongly varies in relation to urbanization and increases with the age of subjects^16,17^. It has been known for a long time that the effects of RhD-genotype are modulated by toxoplasmosis^6,18^. For example, among *Toxoplasma*-free subjects, those who are RhD-negative have extremely good reaction times. However, after *Toxoplasma* infection, the reaction times of RhD-negatives strongly deteriorate. This results in the observation that RhD-negative *Toxoplasma*-infected individuals express the worst reaction times from all subjects. In contrast with that, the reaction times of RhD-positive homozygotes deteriorate only slightly while the reaction times of RhD-positive heterozygotes improve after the *Toxoplasma *infection. It has been suggested that this might show that the natural status of our relatively recent ancestors was actually being *Toxoplasma*-infected with our physiology tuned up to this status^5^. If we continue in this line of thinking, the spreading of the allele for Rh-negativity in Europe could have been related to the relative scarcity of toxoplasmosis in Europe before the advent of the domestic cat—the only important definitive host of *Toxoplasma* in Holocene Europe^6,19^. It is therefore desirable to control for this variable in future studies. The prevalence of latent toxoplasmosis in Czech residents of middle age is relatively high, especially in women^17^. The presence of about one-third of *Toxoplasma*-infected subjects therefore might explain some heterogeneity in the distribution of the health problems score observed in our data (Fig. 1). In future (large-scale) studies, analyses should be done separately for *Toxoplasma*-free and *Toxoplasma*-infected subjects.

### The strengths and limitations of the study

The main strength of the present study is that, for the first time, the RhD-positive subjects were genotyped by a molecular method. In previous studies, either the genotypes were estimated based on the phenotype of parents, or only the effects of Rh phenotypes, not RhD genotypes, were studied. Another important advantage is that the RhD phenotypes/genotypes were estimated in the course of a study and not self-reported by the participants of the study.

The main limitation of the study is that subjects reported their health problems themselves. It is clear that some people might misreport their health problems. However, there is no reason to expect that RhD-positive heterozygotes and homozygotes (mis)reported their problems differently unless they really differ in their health status. It is important to remember that participants were not aware of their *RHD* genotype at the time of filling the questionnaire. Another limitation of the present study is that participants have been self-selected and therefore probably do not represent a typical Czech population. The observed higher prevalence of RhD-negative subjects among the participants (24.2% in women and 27.2% in men) than in the general Czech population (16%) (a phenomenon reported also in all previous Czech studies on volunteers, reviewed in^5^) suggests that RhD-negative subjects have a higher willingness to participate in unpaid scientific studies.

We have no data on the alleles present in the *RHD*-adjacent loci. It is known that a strong linkage disequilibrium exists between these loci^20^. Therefore, we cannot exclude that other genes, e.g., *RHCE* or *SMP1*, not the *RHD*, are responsible for the superiority of *RHD* heterozygotes.

Several rare *RHD* alleles can result in RhD-negative phenotype and *RHD*-positive genotype in a PCR test. The existence of such alleles might have a large clinical importance. However, because of their rarity, they could not influence the results of the present study. The RhD-negative homozygotes were identified by agglutination tests. Theoretically, the presence of a few individuals who will be identified as homozygotes in our genetic test but who will carry one wild and one rare, e.g. a partly deleted allele, can slightly increase the risk of a false negative result in statistical tests—the failure to detect an existing association between *RHD*-genotypes and health. It cannot, however, increase the risk of a false positive result – finding non-existent association. In the future, the study of the health of carriers of such rare alleles could answer the question of whether the zygosity in the *RHD* gene alone or the presence of specific alleles in the *RHD*-adjacent loci are responsible for the observed effects. For such a study, however, the analysis of a much larger data set will be necessary.

## Conclusions

For the first time, we have confirmed the effect of *RHD* genotype on human viability (health) in the sample of subjects directly *RHD*-genotyped by a molecular method. We demonstrated that the effect is modulated by the sex of the subjects—RhD-positive homozygotic women had worse and RhD-positive homozygotic men had better physical health than corresponding RhD-negative homozygotes. These results strongly suggest that the performance of RhD-positive subjects depends on heterozygote/homozygote ratio, which could strongly vary depending on the frequency of the RhD-negative allele in the general population and the method of recruitment of participants to the study. This strongly underlines that the performance and health of subjects with different *RHD*-genotypes, not just phenotypes, should be compared in future studies.

## Materials and methods

### Participants

An invitation to participate in a study “of the effects of genetic and environmental factors on human physiology, personality and behavior” was posted on the timeline of the Facebook page Labbunnies, an approximately 18,000-strong group of Czech and Slovak nationals willing to participate in evolutionary psychology experiments. The participants checked in for the study (for a specific minute) using a web form.

Participants came to our lab where they provided a blood sample for serologic and genetic testing, and completed a set of questionnaires. They received no remuneration for their participation, but obtained the information about their *RHD* genotype, toxoplasmosis seropositivity/seronegativity, and a commemorative badge. Before the blood sampling that occurred at the Faculty of Science in November 2018, the subjects were given written information about the aims and technical details of the study and were asked to sign the informed consent form.

### Questionnaire

On the blood sample taking-day, the subjects were asked to fill in a short questionnaire. They were asked to estimate how many times they had had an acute illness, including an infection, in the past month and the past 6 months (ordinal variables *acute illness in last month* and *acute illness in past 6 months*), how many chronic health problems they had been treated for in the past month (*chronic problems treated now*), and how many other chronic health problems they suffer, including untreated problems (*chronic problems all*), how many kinds of *drugs prescribed* by a medical doctor they were currently taking, how well they felt physically on the blood sample taking-day (*bad physical health today*, 6-points scale 1: excellently, 6: very badly), how often they suffer from *headaches* (1: never, 2: exceptionally, 3: once a month, 4: once a week, 5: daily), and whether they had been diagnosed with tension headaches or *migraines* (0: no, 1: yes). In different questionnaires distributed electronically on another day, they were asked how often they suffer from *allergies*, *skin disorders* (nonallergic), *circulatory system disorders* (heart or vascular), *digestive tract disorders* (ulcer, pancreas, etc.), *orthopedic disorders* (backache, joints ache, etc.), *neurological disorders*, headaches *(headaches 2)*, other *physical pains*, other *chronic* or recurrent *physical problems*, and how frequently they are *tired*. To answer all these questions, participants should use six-points scales (1: never, 2: maximally once a year, 3: several times a year, maximally once a month, 4: several times a month, maximally once a week, 5: two or more times a week, 6: daily or several times a day). They were also asked whether they suffer from *metabolic disorders* (diabetes etc.) (1: definitively no, 2: more likely no, 3: more likely yes, 4: definitively yes), what their *blood pressure* is (1: I do not know—coded as “ND”, 2: very low, 3: rather low, 4: normal, 5: rather high, 6: very high), how often they are *tired* immediately *after* returning from *work* (or school) (1: never, 2: rarely, 3: sometimes, 4: often, 5: nearly always, 6: always), how often they are *tired after* several hours-travel by *train*, (the same scale), and how often they are *tired after* several hours-travel by *bus*, (the same scale). Besides that, they were asked how often they suffer from *common infectious diseases* like influenza or a similar viral or bacterial disease (1: never, 2: maximally once a year, 3: several times a year, maximally once a month, 4: several times a month, maximally once a week, 5: two or more times a week), how often they attend *medical doctors* (not dentist and not for prevention) (1: never, 2: maximally once a year, 3: several times a year, maximally once a month, 4: several times a month, maximally once a week, 5: two or more times a week), how many times they had used *antibiotics in the last year* (1: never, 2: once, 3: twice, 4: three times, 5: four times 6: five or more times), how many times they had used *antibiotics in the last 3 years* (the same scale), how many times they had spent more than a week in a *hospital in the last year* (the same scale), how many times they had spent more than a week in a *hospital in the past 5 years* (the same scale), and how long they expect to live (*life expectancy* 1: more than 99 years. 2: 90–99 years, 3: 80–90 years, 4: 70–79 years, 5: 60–69 years, 6: less than 60 years). *Physical health problems score* was computed as mean Z-score from these 30 physical health-related variables.

In the first questionnaire (before blood taking), participants were asked to rate how well they feel mentally today (*bad mental health today*, 6-points scale anchored with 1: excellent, 6: very bad). In the following electronic questionnaires, they were asked how many specific *learning disabilities* (e.g. dyslexia or dysgraphia) they have, whether they suffer from *phobias* (anchored with 1: no, 6: yes, with many or intensive), from *depression* (1: never, 2 maximally once a year, 3: several times a year, maximally once a month, 4: several times a month, maximally once a week, 5: two or more times a week, 6: daily or several times a day), and from *other mental health problems* (the same scale). They were also asked whether they feel *mentally bad today* (anchored with 1: definitively no, 6: definitively yes), and whether they feel *mentally badly usually* (the same 6-point scale). Separately, participants also completed a standard NEO-PI-R questionnaire with its neuroticism subscales *anxiousness* and *depressiveness*^21^. *Mental health problems score* was computed as a mean Z-score from these 9 mental health-related variables.

### RHD genotyping

RhD-negative homozygotes were identified by a standard agglutination test. A constant amount of anti-D serum (human monoclonal antiD reagent; SeracloneH, Immucor Gamma Inc.) was added to a drop of blood on a white glass plate. Red cells of RhD-positive subjects were agglutinated within 2–5 min.

For discrimination between RhD-positive homozygotes and heterozygotes, we used high-resolution melting technology. For design of PCR primers, we exploited the high but not complete sequence homology between *RHD* and *RHCE* genes exons and introns as well^3^. Therefore, the PCR primers (intron 8 positions in GenBank sequence MH260576.1: 5040–5101 and 9836–9897) were selected from the sites with complete homology to amplify the short sequence (61 bp) with three nucleotide differences inside. This resulted in nearly identical amplification efficiency for both *RHD* and *RHCE* genes and opened the possibility of applying high-resolution melting technology for *RHD/RHCE* copy number determination.

Fresh blood samples (100 µl) were mixed with 200 µl 10 mM Tris buffer of pH 7.0 containing 5 mM EDTA (TE), frozen, and stored in − 20 °C. After thawing, DNA was isolated with phenol–chloroform method and resuspended in the final volume of approximately 50 µl TE. One µl of the sample was added to the 9 µl reaction mix (0.1 µl 5U Taq DNA polymerase (SBD, Tartu, Estland), 1 µl 10× Taq DNA polymerase buffer, 0.6 µl 25 mM MgCl_2_, 0.2 µl 10 mM dNTPs, 0.1 µl primer 5′ CCCAGTGACCCACATGC 3′, 0.1 primer 5′ CCTACATTGTGCTGCTGG 3′, and 7 µl ddH20), mixed and loaded to 96-well plate, overlaid with 10 µl mineral oil and amplified (forty cycles: 95 °C/30 s–56 °C/30 s–72 °C/30 s) in RapidCycler (Idaho Technology, USA). After the amplification, 0.3 µl of LCGreenPlus (Invitrogen, USA), was added to each well and the plate was analyzed on LightTyper (Roche, Switzerland), in temperatures 75–85 °C. One peek at 81 °C was present in the profile of RhD-negative homozygotes and two peeks at 82 °C and 83.5 °C in the profile of RhD heterozygotes (the ratio of heights 2:1) and RhD-positive homozygotes (the ratio of heights 1:1).

### Statistics

R v. 3.3.1^22^ was used for all statistical tests. Associations of *RHD* genotype with sex were measured using a contingency table and the association of these two factors with *physical health problems score* and *mental health problems score* (which had normal distributions) was tested using ANCOVA (Type III sum of squares) with the age of subjects as covariate. Associations of RhD genotype with source health-related variables (which had mostly highly asymmetric distributions) were measured by a (nonparametric) partial Kendall correlation test (R package ppcor 1.1^23,24^) with age as a covariate. Correction for multiple tests was done using the Benjamini–Hochberg procedure with a false discovery rate pre-set to 0.10^25^ using the Excel sheet published by McDonald^26^.

### Ethics

The project was approved by the Ethics Committee of the Faculty of Science, Charles University (No. 2017/08). All experiments were performed in accordance with relevant guidelines and regulations; and informed consent was obtained from all participants and/or their legal guardians.

## Supplementary Information

Supplementary Information.

## Data Availability

All relevant data are available at Figshare—10.6084/m9.figshare.14235884.v1.
