# Supplementary material for: The importance of being heterozygote: effects of RHD-genotype-sex interaction on the physical and mental health of a non-clinical population
Source: Sci Rep. 2021 Nov 9;11:21960. doi: 10.1038/s41598-021-00977-1 (PMC8578618; doi:10.1038/s41598-021-00977-1)
Supplement: Supplementary file 1 — Supplementary Information. [file 41598_2021_977_MOESM1_ESM.docx]

**Supplementary Table 1**. Effects of *RHD* genotype and phenotype on physical and mental health problems scores and their source variables

|  | All | | | | Women | | | | Men | | | |
| --- | --- | --- | --- | --- | --- | --- | --- | --- | --- | --- | --- | --- |
|  | **+-/++** | **+-/--** | **++/--** | p+/p- | **+-/++** | **+-/--** | **++/--** | p+/p- | **+-/++** | **+-/--** | **++/--** | p+/p- |
| *physical health problems score* | 0.095 | 0.959 | 0.131 | 0.457 | **0.027** | 0.328 | **0.006** | 0.053 | 0.968 | 0.070 | 0.040 | 0.035 |
| *mental health problems score* | **0.010** | 0.588 | 0.094 | 0.678 | **0.036** | 0.892 | 0.095 | 0.483 | 0.177 | 0.364 | 0.661 | 0.676 |
| *acute illness in the last month* | 0.991 | 0.138 | 0.179 | 0.119 | 0.670 | 0.196 | 0.410 | 0.218 | 0.554 | 0.577 | 0.268 | 0.362 |
| *acute illness in the past 6 months* | 0.148 | 0.644 | 0.425 | 0.951 | 0.743 | 0.359 | 0.505 | 0.359 | **0.001** | 0.012 | 0.479 | 0.189 |
| *chronic problems treated now* | 0.813 | 0.019 | 0.068 | 0.020 | 0.320 | 0.073 | **0.022** | 0.030 | 0.048 | 0.113 | 0.653 | 0.344 |
| *chronic problems all* | 0.980 | 0.255 | 0.285 | 0.210 | 0.250 | 0.607 | 0.181 | 0.339 | 0.070 | 0.242 | 0.771 | 0.483 |
| *drugs prescribed* | 0.256 | 0.105 | 0.445 | 0.171 | 0.775 | 0.078 | **0.060** | 0.054 | 0.015 | 0.637 | 0.156 | 0.781 |
| *bad physical health today* | 0.368 | 0.101 | 0.307 | 0.126 | **0.057** | 0.157 | 0.769 | 0.429 | 0.247 | 0.485 | 0.036 | 0.168 |
| *headaches* | 0.217 | 0.292 | **0.051** | 0.113 | **0.030** | 0.260 | **0.007** | 0.045 | 0.401 | 0.717 | 0.299 | 0.466 |
| *migraines* | 0.493 | 0.114 | **0.044** | 0.060 | 0.087 | 0.165 | **0.008** | 0.041 | 0.070 | 0.434 | 0.151 | 0.854 |
| *life expectancy* | 0.215 | 0.582 | **0.053** | 0.227 | 0.316 | 0.335 | 0.857 | 0.546 | 0.381 | 0.059 | **0.001** | **0.005** |
| *allergies* | 0.447 | 0.954 | 0.704 | 0.876 | 0.970 | 0.762 | 0.627 | 0.667 | 0.166 | 0.897 | 0.195 | 0.462 |
| *skin disorders* | **0.008** | 0.150 | 0.372 | 0.598 | **0.005** | 0.050 | 0.460 | 0.351 | 0.692 | 0.663 | 0.637 | 0.620 |
| *circulatory system disorders* | 0.710 | 0.795 | 0.647 | 0.682 | 0.283 | 0.578 | 0.585 | 0.969 | 0.444 | 0.412 | 0.997 | 0.514 |
| *blood pressure* | 0.983 | 0.113 | 0.210 | 0.108 | 0.436 | 0.441 | 0.908 | 0.602 | 0.171 | 0.116 | 0.071 | 0.055 |
| *digestive tract disorders* | 0.306 | 0.216 | 0.960 | 0.385 | 0.653 | 0.136 | 0.483 | 0.175 | 0.249 | 0.612 | 0.112 | 0.292 |
| *metabolic disorders* | 0.224 | 0.074 | 0.655 | 0.157 | 0.284 | 0.076 | 0.510 | 0.143 | 0.791 | 0.903 | 0.729 | 0.966 |
| *orthopedic disorders* | 0.127 | 0.233 | **0.015** | 0.056 | **0.028** | 0.165 | **0.002** | 0.021 | 0.793 | 0.449 | 0.546 | 0.463 |
| *neurological disorders* | **0.025** | 0.899 | 0.100 | 0.467 | **0.010** | 0.742 | **0.016** | 0.177 | 0.841 | 0.581 | 0.537 | 0.505 |
| *headaches 2* | **0.044** | 0.185 | **0.001** | 0.021 | 0.101 | 0.022 | **0.001** | **0.002** | 0.186 | 0.151 | 0.690 | 0.435 |
| *physical pains* | **0.008** | 0.913 | **0.031** | 0.372 | **0.003** | 0.924 | **0.019** | 0.338 | 0.386 | 0.703 | 0.485 | 0.922 |
| *chronic physical problems* | **0.000** | 0.042 | **0.053** | 0.643 | **0.000** | 0.210 | **0.001** | 0.465 | 0.933 | 0.076 | 0.064 | 0.034 |
| *tired* | 0.143 | 0.335 | 0.738 | 0.629 | 0.219 | 0.865 | 0.410 | 0.770 | 0.637 | 0.078 | 0.424 | 0.122 |
| *tired after work* | **0.007** | 0.061 | 0.497 | 0.339 | **0.004** | 0.068 | 0.390 | 0.409 | 0.558 | 0.411 | 0.896 | 0.541 |
| *tired after train* | 0.299 | 0.365 | 0.176 | 0.223 | 0.400 | 0.239 | 0.131 | 0.130 | 0.664 | 0.762 | 0.869 | 0.966 |
| *tired after bus* | **0.001** | 0.991 | **0.008** | 0.227 | **0.002** | 0.526 | **0.045** | 0.609 | 0.249 | 0.552 | 0.236 | 0.313 |
| *common infectious diseases* | **0.047** | 0.325 | **0.015** | 0.074 | 0.126 | 0.107 | 0.859 | 0.256 | 0.250 | **0.000** | **0.000** | **0.000** |
| *medical doctors* | 0.137 | 0.602 | 0.090 | 0.260 | 0.076 | 0.307 | **0.018** | 0.077 | 0.889 | 0.534 | 0.550 | 0.531 |
| *antibiotics in the last year* | 0.285 | 0.135 | 0.374 | 0.173 | 0.449 | 0.017 | **0.049** | 0.014 | 0.457 | 0.311 | 0.175 | 0.177 |
| *antibiotics in the last 3 years* | 0.773 | 0.813 | 0.563 | 0.696 | 0.519 | 0.316 | **0.076** | 0.144 | 0.511 | 0.301 | 0.131 | 0.163 |
| *hospital in the last year* | **0.016** | 0.019 | 0.776 | 0.086 | **0.062** | 0.120 | 0.727 | 0.283 | 0.187 | 0.113 | NA | 0.185 |
| *hospital in the past 5 years* | 0.957 | 0.005 | **0.009** | **0.003** | 0.737 | 0.038 | **0.026** | 0.022 | 0.734 | 0.071 | 0.187 | 0.069 |
| *bad mental health today* | **0.014** | 0.566 | 0.162 | 0.788 | **0.005** | 0.908 | **0.023** | 0.320 | 0.850 | 0.335 | 0.318 | 0.285 |
| *learning disabilities* | 0.912 | 0.314 | 0.362 | 0.282 | 0.983 | 0.162 | 0.237 | 0.149 | 0.828 | 0.852 | 0.718 | 0.779 |
| *mentally bad today* | 0.402 | 0.600 | 0.772 | 0.817 | 0.178 | 0.455 | 0.534 | 0.820 | 0.720 | 0.853 | 0.711 | 0.979 |
| *mentally bad usually* | **0.000** | 0.059 | 0.141 | 0.550 | **0.005** | 0.219 | 0.143 | 0.887 | 0.028 | 0.153 | 0.544 | 0.481 |
| *anxiousness* | 0.740 | 0.686 | 0.966 | 0.818 | 0.682 | 0.670 | 0.935 | 0.832 | 0.911 | 0.728 | 0.491 | 0.555 |
| *depressiveness* | **0.049** | 0.370 | 0.415 | 0.822 | 0.197 | 0.277 | 0.932 | 0.505 | 0.143 | 0.889 | 0.277 | 0.676 |
| *phobias* | 0.441 | 0.862 | 0.334 | 0.595 | 0.536 | 0.986 | 0.571 | 0.831 | 0.911 | 0.442 | 0.316 | 0.344 |
| *depressions* | **0.008** | 0.253 | 0.276 | 0.810 | 0.100 | 0.795 | 0.148 | 0.386 | 0.028 | 0.018 | 0.985 | 0.106 |
| *other mental health problems* | **0.033** | 0.140 | **0.002** | 0.017 | 0.185 | 0.098 | **0.012** | 0.028 | 0.067 | 0.844 | 0.061 | 0.309 |

*This table shows the significance of particular effects (p) measured with a nonparametric partial Kendall correlation test controlled for age. The ++, +-, - -, p-, and p+ at the column headings denote RhD-positive homozygotes, RhD-positive heterozygotes, RhD-negative homozygotes, RhD-negative and RhD-positive subjects, respectively. The results that remained significant after the correction for multiple tests with Benjamini-Hochberg procedure are printed in bold.*  *The boarder of significance differs between the columns of the table as it depends not only on the individual p-values but also on the number of significant associations in particular columns.*
